# Translating and Validating the Vietnamese Version of the Health Sciences Evidence-Based Practice Questionnaire

**DOI:** 10.3390/ijerph20075325

**Published:** 2023-03-30

**Authors:** Quyen Thao Nguyen, Mei-Ling Yeh, Ly Thi Hai Ngo, Chiehfeng Chen

**Affiliations:** 1 School of Nursing, National Taipei University of Nursing and Health Sciences, 365 Mingde Road, Taipei City 112, Taiwan; quyennt@ump.edu.vn; 2 Department of Midwifery, Faculty of Nursing and Medical Technology, University of Medicine and Pharmacy at Ho Chi Minh City, 201 Nguyen Chi Thanh Street, Ho Chi Minh City 70000, Vietnam; ngohailya12@ump.edu.vn; 3 Cochrane Taiwan, Taipei Medical University, 252 Wuxing Street, Taipei City 110, Taiwan; clifchen@tmu.edu.tw; 4 Department of Public Health, School of Medicine, College of Medicine, Taipei Medical University, 252 Wuxing Street, Taipei City 110, Taiwan; 5 Division of Plastic Surgery, Department of Surgery, Evidence-Based Medicine Center, Wan Fang Hospital, Taipei Medical University, No. 111, Sec. 3, Xinglong Street, Taipei City 116, Taiwan

**Keywords:** evidence-based practice, Health Sciences-Evidence Based Practice (HS-EBP), midwife, reliability, validity

## Abstract

No validated instrument is available for assessing the evidence-based practice capacity of Vietnamese health professionals. This study aimed to translate and validate the Health Sciences Evidence-Based Practice questionnaire (HS-EBP) from English to Vietnamese and ascertain its psychometric properties. Data were collected from two obstetric hospitals in Vietnam. Participants: A total of 343 midwives were randomly selected. The HS-EBP questionnaire was translated by a group of bilingual experts into Vietnamese (HS-EBP-V). Content validity was assessed by two experts. Internal consistency and test–retest reliabilities were assessed using Cronbach’s α and intraclass correlation (ICC), respectively. Construct validity was assessed using the contrasted groups approach. As a result, the content validity index of the HS-EBP-V reached 1.0. For the individual subscales, Cronbach’s α was 0.92–0.97 and ICC was between 0.45 and 0.66. The validity of the contrasted-groups approach showed discrimination by a significant difference in the subscale scores among diploma holders compared with bachelor’s degree holders (*p* < 0.001). The validation of the HS-EBP questionnaire indicated satisfactory psychometric properties. The results indicate that the HS-EBP is a reliable and valid instrument which assesses the competencies of as well as facilitators of and barriers to the five steps of EBP among midwives. The HS-EBP-V was deemed a reliable and validated tool for assessing the competency and application of EBP among Vietnamese healthcare professionals.

## 1. Introduction

Evidence-based medicine was brought up to emphasise the role of scientific research in clinical decision-making in 1992 [1]. The evidence-based paradigm was then extended to all health professions, in order to encourage them to adopt a critical and objective approach. All healthcare professionals—medical, nursing, and comedical—are required to perform evidence-based practice (EBP). EBP incorporates three components of the best available evidence, practitioners’ clinical expertise, and patients’ expectations [2]. On the basis of this definition, the five essential steps of EBP were described, including formulating answerable questions, finding the best evidence, appraising the evidence, applying them in clinical practice, and evaluating performance [3]. These steps have been the basis for both practising and teaching EBP. They can be applied as a foundation in healthcare systems to improve quality and safety, provide optimal outcomes, and reduce costs [4,5]. Moreover, EBP boosts professional growth and career development and brings job empowerment and satisfaction as it engages healthcare professionals in lifelong learning and builds on professional expertise [6,7,8]. EBP is also inextricably connected to shared-decision making whereby reliable evidence is incorporated into the decision making process, so that patients’ preferences and decisions are evidence-informed. 

Midwives play an important role in health care teams, and attribute direct, independent and autonomous responsibilities in monitoring and coordinating care and treatments [9]. High-quality midwifery care improves over 50 health-related outcomes; however, to improve maternal and neonatal health, strengthening quality midwifery care is required [10]. Therefore, midwives should have competencies to think critically, analyse complex situations, perform health assessment, and make decisions based on research evidence. Gaining the knowledge and skill and displaying a good attitude to EBP is important to make midwives establish and maintain the behaviour of practicing evidence-based care in their profession. Because of these merits, the first Sicily statement outlined that it is a minimum requirement for all healthcare professionals to understand and implement the principles and process of EBP [3]. 

EBP has been recognised as one of the core competencies necessary for the continuous improvement of the quality and safety of healthcare in the United States [11]. Since 2007, the Taiwan National Health Research Institutes has provided EBP-related information resources and promotional activities for healthcare professionals of regional hospitals [12]. Today, the introduction of EBP education in nursing curricula is strongly recommended and even compulsory in many countries such as the United States [13], Australia [14] and United Kingdom [15]. Correspondingly, the Vietnamese Ministry of Health approved EBP as a core component of Vietnamese nursing-midwifery practice in the national competency standards [16]. Although the volume of discussions on EBP has increased exponentially in the last decade in Vietnam, only physicians are familiar with the concept of EBP. In other health-allied professions including midwifery, the term EBP seems obscure and confusing. The importance of maternal and child health is paramount, and the World Health Organization has particularly emphasized the development of the health of mother and baby [17]. Among the various groups involved in the health of the mother and baby, midwives have the highest chance of providing the best care for promoting maternal and baby health [18]. The midwives play a critical role in providing primary care for saving mothers’ life; therefore, improving the quality of midwifery care is paramount. However, in Vietnam, few studies have been conducted in midwifery, and no clinical study has involved midwives. It is critical to have a measurement tool to examine the current situation of EBP among midwives, which will allow appropriate training to be offered to midwives.

EBP competencies refer to four attributes, which are knowledge, attitudes, skills, and performance. Globally, many EBP teaching strategies have been used and evaluated; however, a lack of EBP knowledge and skills as well as inadequate enthusiasm and attitudes are still among the most commonly reported barriers to practising EBP [19,20,21], including among healthcare professionals in Vietnam [22,23]. To increase EBP competencies, continuing medical education is crucial; however, most instruments used in interventional studies are of low quality, and none can evaluate all five EBP steps [24]. The EBP evaluation tools have been discipline-specific and undertaken primarily in the fields of medicine, physical therapy, and most recently, in nursing [25]. Certain questionnaires measure EBP competencies; however, they evaluate only one [26] or two [27] attributes of competencies.

In addition, to assess EBP competencies, performance-based measurements are objective and deliver high quality to reduce bias in self-report questionnaires [25]. Benner asserted that nursing competence assessment should be grounded in real practice within a situational context, under pressure, and over time [28]. Obviously, knowledge tests could be better fit to measures of actual performance of complex tasks such as EBP implementation. Six validated performance-based EBP knowledge and skill measurement tools are available: the Berlin questionnaire [29], Assessing Competency in Evidence-Based Medicine tool [30], Fresno test [31], Evidence-Based Practice Knowledge Assessment in Nursing [32], Taylor’s questionnaire [33], and Utrecht questionnaire [34]. However, none of them measures EBP competencies across all five steps. Although they are validated and relatively objective, the measurements comprise clinical scenarios and open-ended questions, meaning that they are time-consuming and require experts to interpret and grade the answers [25]. No standard exists for performance-based assessment, and the tools have been limited to the field of medicine [30] or nursing [32,35,36].

To overcome these disadvantages, self-reported instruments which have been used to evaluate self-perceived EBP competencies are simpler, cheaper, and more feasible alternatives [37]. The nursing practice questionnaire [38], revised Evidence-Based Practice questionnaire [27], Developing Evidence-Based Practice questionnaire [39], Evidence-Based Practice Beliefs and Implementation Scales [40], Evidence-Based Nursing Attitude questionnaire [41], as well as questionnaires for evidence-based medicine [42] and evidence-based nursing [43] have been developed and used in many studies. However, these questionnaires are not entirely suitable for measuring EBP competencies. They measure neither the three attributes of competence nor the five steps of the EBP process.

A newly developed instrument, the Health Sciences Evidence-Based Practice questionnaire (HS-EBP), addresses the aforementioned shortcomings of the existing tools and can thus evaluate the effects of the EBP’s educational interventions. The HS-EBP questionnaire includes five domains: beliefs and attitudes, results from scientific research, development of professional practice, assessment of results, and barriers and facilitators [44]. The components of the domain of results from scientific research include the first three steps of the EBP process: the formulation of clinical questions, search for and recovery of the best evidence, and critical appraisal. The domain of development of professional practice corresponds to step 4, which is a professional’s ability for individual judgement and actions, and the preferences of patients as well as the use of other potential sources of information for decision-making. The domain of assessment of results constitutes the last step, which is the evaluation of effects on professional practice and healthcare outcomes. These three domains evaluate the knowledge and skills, especially in EBP application, of health professionals. The beliefs and attitudes domain reflects the professionals’ beliefs and attitudes in relation to EBP. The barriers and facilitators domain refers to the organisational aspects or context structure in which practice takes place and constitutes a determining element for implementing EBP in clinical settings. The domains of beliefs and attitudes and barriers and facilitators influence all the steps of the EBP process. The HS-EBP questionnaire evaluates EBP competencies and its application across the healthcare discipline as it considers EBP in a transdisciplinary way [45]. However, no Vietnamese translation of the HS-EBP questionnaire is available; this hinders the evaluation of EBP competencies and its appropriate and comprehensive application in the Vietnamese population. This study aimed to translate the HS-EBP questionnaire from English to Vietnamese and ascertain its psychometric properties.

## 2. Methodology

This study had two phases: the translation of the HS-EBP questionnaire and the assessment of its reliability and validity.

### 2.1. Phase I: Forward and Back Translating the HS-EBP Questionnaire 

After obtaining permission from the developers [44], the HS-EBP questionnaire was translated from the original English language into Vietnamese by using forward and backward translation [46]. The linguistic translation was performed using a decentred strategy to ensure the meaning and meaning equivalence in the two cultures. Therefore, first, the original English version of the HS-EBP questionnaire was independently translated to Vietnamese by three translators, including one English teacher (NT), one nursing lecturer (PH), and one obstetrician (NT). Second, the three translated versions were compared and contrasted by a team (ML, NT, MD, and CH) to reach a draft agreement of the meaning in the Vietnamese version of the HS-EBP questionnaire. Third, the Vietnamese version was independently back-translated into English by other two translators (NT and TA) blinded to the original questionnaire, and this version was compared with the original English version by a native English-speaking senior instructor (GC). Any significant discrepancy detected was addressed so that the Vietnamese version would have identical meaning to the original version. All the translators involved were bilingual in Vietnamese and English and familiar with both cultures. Finally, in-depth cognitive interviews were conducted to assess participants’ understanding of the questions and specific terms and to identify difficulties with the response choices. This approach allowed us to make sure that the translated items retained the same meaning as the original items [47]. The completed version of the HS-EBP questionnaire was assessed using a pilot sample of 17 midwives to ensure understanding of the intended meaning of each item. A researcher conducted a verbal cognitive interview, asking the respondents to elaborate what they thought of each questionnaire item and their corresponding response. After this test, the Vietnamese version of the HS-EBP questionnaire (HS-EBP-V) was finalised.

### 2.2. Phase II: Validating the HS-EBP-V

#### 2.2.1. Participants

Two qualified nurse teachers who teach EBP evaluated the content validity of the questionnaire. Considering the respondent-to-item ratio of 5:1, a minimum sample of 300 midwives was required [48]. Midwives with more than one year of clinical experience and working at either of two obstetrics and gynaecology hospitals in Ho Chi Minh City, Vietnam, were invited and randomly selected from department personnel directory. Of them, 30 midwives completed the HS-EBP-V twice at an interval of 2 weeks to assess test–retest reliability. Participants’ demographic characteristics included age, marital status, educational level, working department, years of working experience, EBP-related learning experience, and English competence level.

#### 2.2.2. Research Design

This study validated the HS-EBP questionnaire based on the measurement theory [46].

#### 2.2.3. Measurements

The original 60-item HS-EBP questionnaire included five subscales: beliefs and attitudes, results from scientific research, development of professional practice, assessment of results, and barriers and facilitators [44]. The questionnaire was developed using a modified Delphi technique based on a heterogeneous group of 32 experts encompassing nurses, physiotherapists, physicians and psychologists [45]. Each item was rated on a 10-point Likert scale (ranging from 1 to 10), with higher scores indicating a greater degree of agreement. The components of the domain of results from scientific research include the first three steps of the EBP process: the formulation of clinical questions, search for and recovery of the best evidence, and critical appraisal. The domain of development of professional practice corresponds to step 4, which is a professional’s ability for individual judgement and actions, and the preferences of patients as well as the use of other potential sources of information for decision-making. The domain of assessment of results constitutes the last step, which is the evaluation of effects on professional practice and healthcare outcomes. These three domains evaluate the knowledge and skills, especially in EBP application, of health professionals. The beliefs and attitudes domain reflects the professionals’ beliefs and attitudes in relation to EBP. The barriers and facilitators domain refers to the organisational aspects or context structure in which practice takes place and constitutes a determining element for implementing EBP in clinical settings. The domains of beliefs and attitudes and barriers and facilitators influence all the steps of the EBP process. The questionnaire demonstrates sufficient measurement properties, in terms of internal consistency with Cronbach’s alpha ranging from 0.84 to 0.96, construct validity and criterion validity [44]. 

#### 2.2.4. Procedures

After randomly selecting the midwives, the researchers approached the midwives at their workplaces, explained the research aims, requested their consent for participation in the study, and asked them to fill the electronic version of HS-EBP-V and a sociodemographic survey through Google Forms. The request for research participation came from a researcher who had no control over the work performance of hospital staff. Thus, the recruitment strategy was perceived by the participants as not coercive. Participants were then randomly selected to participate in a cognitive interview. The cognitive interview further investigated the content validity of the HS-EBP-V by providing insight into what the respondents actually reflected upon while answering the questionnaire. 

#### 2.2.5. Data Analysis

The item-objective relevance to determining content validity was used to determine the item-level content validity index (I-CVI) and instrument-level content validity index (S-CVI) [44]. The CVI was calculated using the ratio of scores 3 and 4 to the total number of experts. I-CVI levels of ≥0.78 and S-CVI levels of ≥0.90 are recommended [49]. Cronbach’s alpha was used to estimate internal consistency reliability; a value of at least 0.70 has been suggested to indicate adequate internal consistency [50]. ICC was used to calculate test–retest reliability, and its 95% confidence intervals were calculated based on consistency and a two-way mixed-effects model. To ascertain the extent to which the two sets of scores were correlated, an ICC value of <0.5 indicated poor reliability; an ICC value of 0.5–0.74 indicated moderate reliability; and ICC value of 0.75–0.9 indicated good reliability; and an ICC value of >0.9 indicated excellent reliability [51]. Construct validity was determined using a contrasted groups approach and the independent t-test, in which the top 160 scores of 342 study participants were divided into two groups by education levels, and diploma and bachelor’s degree holders (*n* = 80 each) [46]. Data are presented as the mean and standard deviation (SD) or frequencies and percentages. All statistical analyses were conducted using SPSS, version 26.0 (SPSS, Chicago, IL, USA), and *p* < 0.05 was considered statistically significant.

#### 2.2.6. Ethical Consideration

All participants were provided a detailed explanation of the study purpose and procedures, and written informed consent was obtained before data collection. Anonymity was maintained by assigning code numbers to each answer sheet. Participation in the study was voluntary, and the participants could withdraw from the study at any time without having it affect their work.

## 3. Results

### 3.1. Participants

A total of 342 questionnaires were distributed and collected. A high response rate and completeness of the data was observed (100%). Table 1 presents the participants’ demographic characteristics. The mean age was 35.49 (SD = 7.57) years, and the mean number of years of professional working experience was 12.34 (SD = 7.33) years. Nearly 50% had a bachelor’s degree. A large proportion worked in inpatient departments (*n* = 248, 72.51%) and reported having a professional licence (*n* = 338, 98.83%). Most midwives were married (*n* = 236, 69.01%) and had multiple children (*n* = 135, 39.47%). In addition, the mean level of reading difficulty of English articles was 6.84 (SD = 2.19). Approximately half of the participants (*n* = 187, 54.68%) learned about EBP when studying at school. Most participants had no previous experience in asking an answerable clinical question (*n* = 153, 44.74%), searching studies (*n* = 211, 62%), or appraising research articles (*n* = 274, 80.12%). More than half of the participants had learned how to apply EBP in clinical practice (*n* = 206, 60%) and had experience in applying EBP in clinical practice (*n* = 180, 52.63%).

### 3.2. Reliability and Validity for the HS-EBP

Table 2 summarises the results of the HS-EBP-V subscales and shows that the mean standardised scores ranged from 83.31 (SD = 12.44) to 89.44 (SD = 9.47). Cronbach’s alpha for the internal consistency of the five subscales was 0.92–0.97, indicating good internal consistency. The ICC for the HS-EBP-V was 0.45–0.66, indicating low to moderate stability. The I-CVI and S-CVI were 1.00, which indicated the high content validity of the items the of HS-EBP-V. 

Table 3 presents the results of the independent t-test for the validity of the contrasted-groups approach; the mean scores in four subscales were significantly higher among bachelor’s degree holders than diploma holders (*p* < 0.001).

### 3.3. Cognitive Interview

Cognitive interviews were administered to 17 participants, with an average duration of 60 min each to ensure understanding of the intended meaning of each item in the HS-EBP-V. The results revealed that the interviewees had difficulty in answering items containing terms such as evidence-based practice, study design, PICO question, methodology, and database because of the lack of knowledge on EBP. Modifications to fix these problems were not made for these terminologies, but the EBP training programme should be conducted in the future to improve EBP competencies.

## 4. Discussion

This study translated the HS-EBP questionnaire from the original English language into Vietnamese by using forward and backward translation. The psychometric properties of the HS-EBP-V yielded satisfactory reliability and validity. This is the first study to provide the Vietnamese version of the HS-EBP questionnaire. First, the HS-EBP-V demonstrated good content validity and had five domains: beliefs and attitudes, results from scientific research, development of professional practice, assessment of results, and barriers and facilitators for Vietnamese midwives. These findings were consistent with those of Fernández-Domínguez et al. in which the best fit corresponded to the five domains [44]. Most items of the HS-EBP-V were relevant to their own domain objectives according to all experts’ consensus in relation to the level of the item, and the computed scale relevance, as CVI was excellent. As Fernández-Domínguez et al. claimed, the questionnaire was well designed, applied the utmost methodological rigour, and provided a connection between theoretical claims and empirical data [44]. In addition, construct validity in terms of the contrasted groups approach in this study suggested good validity. 

The HS-EBP-V is the first measure of EBP competency and application in Vietnamese which covers the five steps of the EBP process that correlate with each other. The existing Vietnamese scales of EBP did not target the entire EBP process, such as EBP knowledge [52], readiness for EBP, barriers to EBP scales [53], and EBP attitudes and beliefs [54]. It has been argued that proficiency in critical appraisal may not be an essential pre-requisite for EBP, and thus it may not be necessary to evaluate this step of the EBP process [55]. The instrument developed by McCluskey and Bishop has scoring weighted differently among the steps, as the developers supposed that the emphasis was placed on making the focus of the PICO questions clear to find the most appropriate articles to inform practice [56]. Tilson stressed the importance of including assessment of the application and auditing step [57]. However, the content validity of EBP questionnaires has been critiqued for the inability to measure the entire EBP process [24,25]; hence, these are unable to provide valuable insights into EBP competencies for nurse-midwives. As all dimensions of EBP are covered by the measurements, the five-step EBP process would be assessed more comprehensively. Note that EBP competencies may differ across healthcare professions with contrasting experience levels, such as novice versus expert learners [58]. 

The HS-EBP-V demonstrated excellent internal consistency and reliability, which is consistent with other versions [8,44]. This indicates the consistency across HS-EBP-V items. In general, all the HS-EBP questionnaire items reflected the same underlying EBP construct, suggesting that the Vietnamese version is reliable. Notably, the stability of the HS-EBP-V was confirmed within a 2-week interval, with poor reliability observed in the barriers and facilitators subscale and moderate reliability in the other subscale. This is similar to the findings reported for the original HS-EBP questionnaire [44]. This problem has emerged from the initial version in the stage in which the domain structures and key contents of the questionnaire were developed [45]. The questionnaire developers suggested a better operational definition of these attributes. For example, barriers/facilitators could be the description of the organisational aspect or context structure in which the practice takes place [59], or of supportive cultures for EBP [44]. The EBP process is complex [3], and the related barriers and facilitators are also dynamic. Barriers to EBP implementation are also related to individual aspects, including a lack of time to read the literature, insufficient proficiency in the English language, a lack of ability to work with computers, and a lack of autonomy to change practice [60]. This makes the operationalisation more difficult. Another explanation for this poor reliability may be related to the study sample, which comprised only midwives. A sample that is too homogenous may lead to low variability in the subscale scores [61]. In addition, certain background characteristics, such as EBP experience, may also have contributed to the poor stability. The participants in this study felt that the questions were repetitive; thus, some respondents, perhaps due to a lack of EBP background knowledge, could not differentiate between items. In other words, they may have interpreted items in the same manner and provided the same answers, but they may have not maintained stability in their interpretation and answers across time. To confirm the optimal stability of the reliability of the HS-EBP-V, further research on EBP competency and application is warranted.

The validity of the contrasted groups approach was demonstrated as the HS-EBP-V discriminated between midwives with diplomas and bachelor’s degree in terms of EBP competencies and applications in this study. This is similar to the findings reported for the original HS-EBP questionnaire [8,44]. This is because in Vietnam, EBP is included in bachelor of nursing-midwifery programmes. EBP must be prioritised in all nursing-midwifery curricula and in the context of continuing education to help nurses and midwives develop EBP competencies as well as positive attitudes and beliefs, which are relevant for its application in routine clinical practice. Nowadays, EBP is the global practical model for international healthcare providers and interprofessional collaboration; therefore, EBP empowers, inspires and supports nurse-midwives to maintain their professional development and practice worldwide. On average, the participants’ scores in this study were higher across all the subscales than those from previous studies [8,44]. This is because the nurse-midwives in this study perceived less barriers in their working environments and were encouraged to apply EBP as well. Nurse-midwives used to make decisions based on their traditional clinical experiences. A paradigm shift is now starting to arise as nurse-midwives are introduced to and easily have access to research, conference, seminars, so that they perceive and acknowledge that scientific information is meaningful to themselves and patients. Additionally, clinicians are encouraged to engage in research and quality improvement projects to meet the criteria for professional practice according to the Vietnam Quality Hospital Quality Index 2016 promulgated by the Ministry of Health [62]. Another reason for the scores could be social desirability and the tendency to rush to complete an online survey [63]. Moreover, in Vietnamese culture, the participants may also mark-up answers because they may be afraid of findings that might show the limitations of an organisation [64]. 

## 5. Strengths and Weaknesses

This study applies methodological theories to guide the transparent translation, cross-cultural adaption, evaluation, and reporting of measurement properties. Moreover, the random sampling method and the high response rate help reduce the sampling bias. This sample size was adequate for evaluating internal consistency, test–retest reliability, and the validity of the contrasted groups approach. This is the first study to validate a questionnaire measuring Vietnamese midwives’ EBP competencies and applications. 

This study also has some weaknesses. First, all midwives were recruited from only two hospitals, thus limiting the generalisability of the study findings. Second, participants may have overestimated their actual competencies and applications in the self-reported online questionnaires. Moreover, assessing EBP competence and application in all five EBP steps with one instrument remains a challenge. Self-perception questionnaires such as the HS-EBP questionnaire join the debate surrounding scores obtained using more objective instruments in order to measure actual performance in different clinical settings. Finally, all participants were female, which may have introduced a sex bias.

## 6. Conclusions 

The HS-EBP-V is a reliable and valid tool for effectively assessing EBP competencies among and its application by Vietnamese healthcare professionals and identifying barriers and facilitators of EBP. Furthermore, the HS-EBP-V can be used in research to evaluate the impact of efforts of implementing and maximising EBP. Indicating the deficient knowledge and skills can help in designing educational curricula. The HS-EBP-V allows assessment on the individual and organisational levels. On the individual level, self-evaluation by using this questionnaire can help professionals determine their limitations in EBP competence that influence the process of clinical reasoning and decision-making, such as the beliefs and attitudes of professionals towards EBP. On the organisational level, it can highlight barriers to and facilitators and EBP application, thereby allowing organisations to identify and address the issues, including providing resources or training on EBP to their staff. Information gathered from the administration of the HS-EBP-V can assist policymakers in identifying the level of knowledge, practice, and barriers of EBP and in improving its uptake in clinical practice.

The HS-EBP-V can be used as a validated questionnaire to assess EBP competence on the individual level, especially for Vietnamese midwives. More studies should be conducted on this questionnaire with many different professional roles and types of evidence to confirm its validity in clinics and interventions. Further research should involve hospitals of different levels to expand the generalisability of the results. A more sex-balanced cohort including male practitioners should also be obtained. The validity of the HS-EBP-V questionnaire can be approved by conducting an intervention study not only for midwives but also for other healthcare professionals.

## Figures and Tables

**Table 1:** Demographic characteristics of the participants (*n* = 342).

Variables	*n*	%	Mean	SD
Age (years)			35.49	7.60
Hospital				
A/B	63/279	18.42/81.58		
Working department				
Inpatient/Outpatient	248/94	72.51/27.49		
Marital status				
Married/Single	236/106	69.01/30.99		
Education level				
Diploma/Bachelor’s Degree	173/169	50.58/49.42		
Position				
Staff/Administrator	289/43	84.50/15.49		
Year of working experience			12.34	7.33
Job satisfaction			8.74	1.06
Self-assessed quality of midwifery care			8.32	0.97
Language barrier to scientific literature			6.84	2.19
Learning EBP at schooling				
No/Yes	155/187	45.32/54.68		
Learning EBP in hospital				
No/Yes	200/142	58.48/41.52		
Learning experience: PICO				
No/Yes	153/189	44.74/55.26		
Learning experience: Searching				
No/Yes	211/131	61.70/38.30		
Practice experience: Searching				
No/Yes	203/139	59.36/40.64		
Learning experience: Appraisal				
No/Yes	227/115	66.37/33.63		
Practice experience: Appraisal				
No/Yes	274/68	80.12/19.88		
Written an evidence-based report				
No/Yes	312/30	91.23/8.77		
Learning experience: Applying				
No/Yes	136/206	39.77/60.23		
Practice experience: Applying				
No/Yes	162/180	47.37/52.63		

**Table 2:** Summary results of the HS-EBP-V subscales (*n* = 342).

Subscales	Mean	SD	Alpha	ICC	95% CI
Beliefs and attitudes	89.44	9.47	0.94	0.66	0.41–0.81
Results from scientific research	83.31	12.44	0.97	0.58	0.27–0.75
Development of professional practice	85.90	10.96	0.92	0.64	0.38–70.9
Assessment of results	86.14	11.69	0.97	0.51	0.21–0.72
Barriers and facilitators	84.21	12.25	0.95	0.45	0.13–0.67

SD, standard deviation; ICC, the intraclass correlation coefficient; CI, confidence interval.

**Table 3:** Contrasted group validity for the subscales.

Subscales	Diploma (*n* = 80) Mean (SD)	Bachelor (*n* = 80) Mean (SD)	*t (p)*
Beliefs-attitudes	81.12 (8.78)	95.62 (4.74)	−12.99 (< 0.001)
Results from scientific research	73.49 (9.40)	92.63 (5.90)	−15.40 (< 0.001)
Development of professional practice	77.15 (9.45)	92.94 (6.00)	−12.61 (0.02)
Assessment of results	76.46 (9.39)	94.67 (4.85)	−15.39 (< 0.001)
Barriers-facilitators	74.38 (8.91)	92.84 (5.95)	−15.39 (< 0.001)

SD, standard deviation; independent *t*-test.

## Data Availability

The data that support the findings of this study are available from the corresponding author, upon reasonable request.
